# A GntR-Like Transcription Factor HypR Regulates Expression of Genes Associated With L-Hydroxyproline Utilization in *Streptomyces coelicolor* A3(2)

**DOI:** 10.3389/fmicb.2019.01451

**Published:** 2019-06-26

**Authors:** Magdalena Kotowska, Michał Świat, Justyna Zarȩba-Pasławska, Paweł Jaworski, Krzysztof Pawlik

**Affiliations:** Hirszfeld Institute of Immunology and Experimental Therapy, Polish Academy of Sciences, Wrocław, Poland

**Keywords:** *Streptomyces coelicolor*, regulation of gene expression, L-hydroxyproline, GntR-like protein, FadR subfamily, zinc-binding protein

## Abstract

Bacteria from the genus *Streptomyces* have been long exploited as the most prolific producers of antibiotics, other secondary metabolites and enzymes. They are important members of soil microbial communities that can adapt to changing conditions thank to the fine regulation of gene expression in response to environmental signals. *Streptomyces coelicolor* A3(2) is a model organism for molecular studies with the most deeply recognized interactions within the complex metabolic and regulatory network. However, details about molecular signals recognized by specialized regulatory proteins as well as their direct targets are often missing. We describe here a zinc-binding protein HypR (SCO6294) which belongs to FadR subfamily of GntR-like regulators. The DNA sequence 5′-TACAATGTCAC-3′ recognized by the HypR protein in its own promoter region was identified by DNase I footprinting. Binding of six DNA fragments containing similar sequences located in other promoter regions were confirmed by the electrophoretic mobility shift assay (EMSA). The sequences of 7 *in vitro*-determined binding sites were assembled to generate a logo of the HypR binding motif, 5′-CTNTGC(A/C)ATGTCAC-3′. Comparison of luciferase reporter genes expression under the control of cloned promoter regions in *S. coelicolor* A3(2) wild type and deletion mutant strains revealed, that the HypR protein acts as a repressor of its target genes. Genes belonging to the regulon of HypR code for enzymes putatively involved in collagen degradation and utilization of L-hydroxyproline (L-Hyp) as concluded from predicted structure and conserved domains. Their transcription is induced in the wild type strain by the addition of L-Hyp to the culture medium. Moreover, knockout of one of the genes from the predicted L-Hyp utilization operon abolished the ability of the strain to grow on L-Hyp as a sole source of carbon. To our knowledge, this work is the first indication of the existence of the pathway of L-hydroxyproline catabolism in Streptomycetes.

## Introduction

Morphological differentiation and secondary metabolism of Streptomycetes is controlled by a complex regulatory network (Liu et al., 2013). A variety of transcription factors respond to changing environmental conditions by adjusting gene expression. The chemical signals which influence gene expression include available nutrients (or lack of them), metal ions, toxic compounds and signaling molecules produced by other cells of the same organism or by other organisms (van der Heul et al., 2018). One of the driving forces of the studies of the regulatory mechanisms is the hope to increase titers of known useful compounds and to find ways to “wake up” cryptic genes for unknown natural products which may potentially become new drugs (Zarins-Tutt et al., 2016). *Streptomyces coelicolor* A3(2) has been a model for genetic studies of the genus *Streptomyces* for over five decades (Hopwood, 1999). It produces four pigmented compounds of polyketide origin: blue actinorhodin, red undecylprodigiosin, gray pigment of spores and yellow coelimycin. A number of regulatory proteins governing the molecular mechanisms of secondary metabolism and differentiation of this bacterium have been identified (Flärdh and Buttner, 2009; Van Keulen and Dyson, 2014). Nevertheless, the characterized regulators compose only a fraction of nearly a thousand of potential regulatory proteins coded by the *S. coelicolor* A3(2) genome (Bentley et al., 2002).

Bacterial transcription factors from GntR family regulate gene expression in response to environmental signals such as availability of different carbon sources including complex food sources such as chitin. Some of them are pleiotropic regulators, which may act as both repressors and activators of diverse metabolic pathways. They control primary metabolic processes maintaining the balance of specific compounds and are involved in interconnecting the primary and secondary metabolic pathways. They bind DNA by N-terminal winged helix-turn-helix (WHTH) domains and their activity is modulated by binding ligands. The diversity of C-terminal ligand binding domains is the basis for classification of GntR-like proteins into seven subfamilies (FadR, HutC, MocR, YtrA, AraR, DevA, and PlmA) (Hoskisson and Rigali, 2009).

FadR subfamily is the most abundant group of bacterial GntR-like regulators. FadR from *Escherichia coli* acts as a switch coordinating fatty acid biosynthesis and β-oxidation. When bound to DNA it represses fatty acid degradation (*fad*) genes and promotes transcription of *fabA* gene required for biosynthesis of unsaturated fatty acids. Binding of a long chain acyl-CoA effector molecule releases FadR protein from DNA, leading to de-repression of *fad* genes and inactivation of *fabA* (Xu et al., 2001). Other members of this subfamily often control transport and catabolism of amino acids and other organic acids.

*S. coelicolor* A3(2) genome contains 56 genes for GntR-like proteins representing five subfamilies. Eleven of them were described experimentally (Hoskisson et al., 2006; Rigali et al., 2008; Horbal et al., 2013; Persson et al., 2013; Cen et al., 2016; Yu et al., 2015, 2016; Tsypik et al., 2016, 2017), but the low molecular ligands have been identified in only two cases. Glucosamine 6-phosphate is the effector molecule bound by a master regulator DasR which links nutrient stress to antibiotic production (Rigali et al., 2008). The repressor of gluconoate operon, GntR (SCO1678), was recently shown to respond to both gluconoate and glucono-1,5-lactone (Tsypik et al., 2017). In the current work we characterized a GntR-like protein HypR (SCO6294) from *S. coelicolor* A3(2) and identified its regulatory targets forming putative L-hydroxyproline degradation pathway.

## Materials and Methods

### DNA Manipulation and Bacterial Strains Growth Conditions

DNA manipulations were carried out by standard protocols (Sambrook and Russell, 2001). All the PCR amplified fragments were first cloned into p-GEM-T Easy vector (Promega) or pTZ57R/T (Thermo Fisher Scientific), verified by DNA sequencing and cloned into appropriate plasmids. Primers, as well as bacterial strains and plasmids, are listed in Supplementary Tables S1, S2, respectively. Culture conditions, transformation and conjugation methods followed the general procedures for *E. coli* (Sambrook and Russell, 2001) and *Streptomyces* (Kieser et al., 2000). *S. coelicolor* was cultivated on the following media: modified 79 medium (without glucose) (Pawlik et al., 2010) and MS (Kieser et al., 2000). For positive selection in bacterial one-hybrid system NM medium was used (Meng et al., 2005). The ability to utilize L-Hyp (50 mM) as a sole source of carbon was tested in the liquid Minimal Medium with (NH_4_)_2_SO_4_ as a nitrogen source (Kieser et al., 2000).

### Bacterial One-Hybrid System

A one-step selection procedure of Meng et al. (2005) was applied to isolate clones containing the sequences recognized by HypR protein. The *hypR* gene was PCR amplified using the primer pair B1 and B2 and cloned into NotI and BamHI restriction sites of pB1H1 plasmid, giving pB1H1-SCO6294, so that HypR was expressed as a fusion protein with the α-subunit of RNA polymerase. Selection strain *E. coli* US0 (Δ*hisB*Δ*pyrF*) (Meng et al., 2006) containing plasmid pB1H1-SCO6294 was transformed with a purified library pH3U3-18random (Wolański et al., 2011). Positive clones were selected on plates with NM medium with 5 mM 3-AT (3-amino-triazole, a competitive inhibitor of HIS3 that provides selection for active promoter), kanamycin (30 μg/ml) and chloramphenicol (34 μg/ml). Fragments of pH3U3 plasmid covering the randomized 18 bp sequences from selected clones were amplified using the primer pair B3 and B4 and sequenced. The sequences were analyzed using the MEME algorithm (Bailey and Elkan, 1994)^1^.

### Construction of *hypR* and *SCO6293* Deletion Strains

The *hypR* gene was deleted by two rounds of homologous recombination (Kieser et al., 2000). Flanking arms were amplified (Supplementary Figure S4) and cloned into pOJ260 plasmid. In the first round the gene was replaced with hygromycin resistance cassette (Ω*hyg*) using the plasmid pJZ14 resulting in strain P130. In the second round Ω*hyg* cassette was removed using the plasmid pJZ15 to obtain strain P138. Deletion was verified by PCR (Supplementary Figure S4). *SCO6293* gene was deleted using homologous recombination facilitated by SceI meganuclease (Fernández-Martínez and Bibb, 2014). Left and right flanking arms were amplified and sequentially cloned into pIJ12738 vector resulting in pMK44 plasmid (Supplementary Table S2). pIJ12742 plasmid was used to deliver I-SceI meganuclease. Exconjugants after single and double crossing over events were verified by PCR with D12 and D13 primer pair. The SCO6393 deletion mutant strain was named P201.

### Construction of Expression Plasmids

*hypR* gene was amplified using the primer pair G1 and G2 and cloned into NdeI and HindIII sites of pET28a(+) vector to give plasmid pJZE1 for expression of His-tagged protein and into BamHI and HindIII sites of pET28NStrep vector to give plasmid pMS08 for expression of Strep-tagged protein.

### Purification of His-Tagged *HypR* Protein

*E. coli* BL21(DE3)pLysS strain containing pJZE1 was grown in LB medium with kanamycin (30 μg/ml) and chloramphenicol (34 μg/ml) to an OD_600_ of 0.4–0.5 at 30°C. Expression was induced with 0.1 mM isopropyl-b-D-thiogalactopyranoside (IPTG) at 30°C for 4 h. After harvesting by centrifugation, the cells from 1 l culture were lysed in 40 ml of BWS-10 buffer (100 mM Tris-HCl pH 8.0, 400 mM NaCl, 10 mM imidazole) by sonication and centrifuged at 4°C, 26,890 × g, 30 min. The clarified lysate was supplemented with 2% Tween 20, mixed with 0.5 ml of Co^2+^ loaded Chelating Sepharose Fast Flow resin (Amersham Biosciences) and gently agitated for 30 min at room temperature. The resin was collected by centrifugation (4°C, 500 g, 5 min) washed with BWS-10 and transferred to a column. His-tagged HypR protein was eluted by increasing concentration of imidazole in BWS buffer (40 and 250 mM). The eluted fractions were examined using SDS-PAGE. Equal volume of glycerol was added to the fractions (to the final concentration of 50%) and the protein was stored at −20°C.

### Purification of Strep-Tagged HypR Protein

*E. coli* BL21(DE3) strain containing pMS08 was grown in 1 l LB medium with kanamycin (30 μg/ml) to an OD_600_ of 0.7 at 37°C. Expression was induced with 0.1 mM IPTG at 30°C for 4 h. Cells harvested by centrifugation were disrupted by sonication in 10 ml of buffer W (100 mM Tris-HCl pH 8.0, 150 mM NaCl) and centrifuged at 4°C, 26,890 × g, 30 min. The clarified lysate was mixed with 1 ml of Strep-Tactin Superflow high capacity resin (IBA). The resin was transferred to an empty column and washed with buffer W (7 ml), buffer W supplemented with 200 mM MgCl_2_ (4 ml) and buffer W (8 ml). Strep-tagged HypR protein was eluted with buffer E (buffer W supplemented with 2.5 mM desthiobiotin). Eluted protein was stored at 4°C until use.

### Electrophoretic Mobility Shift Assay (EMSA)

Amplified promoter fragments (see Supplementary Table S1) were cloned directly into the pTZ57R/T vector and used as templates for amplification with IRDye labeled primers P10 and P11 complementary to the sequences flanking the cloning site of pTZ57R plasmid. Each EMSA sample contained 0.03 pmoles of labeled DNA in 16 μl binding buffer (10 mM Tris-HCl pH 7.5, 50 mM KCl, 2.6 mM DTT, 0.16% Tween20, 6 ng/μl herring sperm DNA). His-tagged or Strep-tagged HypR protein was added and samples were incubated for 20 min at room temperature. Four microliter of 40% sucrose was added and samples were loaded on nondenaturing 4% polyacrylamide–Tris–borate–EDTA gels. The results were visualized on Typhoon FLA 9500 apparatus. To test the effect of metal ions on the binding of DNA respective metal chlorides solutions were added at the concentration 5 mM.

### DNase I Footprint

For footprinting experiment the fragment p6294 was uniquely radiolabeled at one end during amplification with the primer pair P3 and P4. One of the primers was labeled on its 5′ end with [^32^P]-ATP using T4 polynucleotide kinase. A binding mixture contained the radiolabeled fragment (approximately 200) in 20 μl binding buffer (10 mM Tris-HCl pH 7.5, 50 mM KCl, 1 mM DTT) and a variable amount of His-tagged HypR protein. The samples were incubated for 20 min at 25°C and 10 min at 30°C. For digestion, 2.5 μl of 10× reaction buffer with MgCl_2_ for DNase I (Fermentas, Thermo Fisher Scientific) and 2.5 μl of DNase I diluted in 1× reaction buffer (0.005 u/μl) were added and samples were incubated for exactly 5 min at 30°C. The reaction was stopped by addition of 25 μl of Stop buffer (200 mM NaCl, 100 mM EDTA, 1% SDS) and incubation for 10 min at 75°C. DNA from each sample was extracted with phenol/chloroform/isoamyl alcohol (25:24:1) and ethanol-precipitated. The precipitated DNA was dissolved in loading buffer (95% formamide, 20 mM EDTA, 0.05% bromophenol blue, 0.05% xylene cyanol FF), loaded onto a denaturing 8% polyacrylamide –Tris–borate–EDTA gel and visualized with a Typhoon FLA9500 Variable Mode Imager (GE Healthcare). Dideoxy sequencing ladders were generated using Thermo Sequenase Cycle Sequencing Kit (Affymetrix) and the same labeled primers as those used to prepare the probes.

### Tryptophan Fluorescence Measurements

Metal ion binding was assayed by monitoring of tryptophan fluorescence (λ_ex_ = 278 nm, λ_em_ = 335 nm) in a 96-well black polystyrene plate on a microplate reader Synergy H4 Hybrid Reader (BioTek). One hundred and fifty microliter samples containing 4 μM Strep-tagged HypR protein in a buffer (100 mM Tris-HCl pH 7.5, 150 mM NaCl) were titrated with 37.5 μM solutions of metal chlorides in 2 μl increments up to the concentration 13 μM in the sample. The data for Zn^2+^ binding were fitted by DynaFit programme (Kuzmič, 1996) to the appropriate chemical model (2:1; metal to protein) with metal–buffer interactions included (logK_ZnTris_ = 2.27; logK_NiTris_ = 2.67) (Zheng et al., 2009).

### Luciferase Reporter Gene Activity Assay

DNA fragments containing potential promoter regions were PCR amplified using primers listed in Supplementary Table S1, and cloned into the reporter plasmid pFLUXH. The fragments p5911/p5912 and p7176/p7177 were cloned into NdeI site and clones in both orientations were identified by PCR with P9 starter complementary to the sequence outside of the cloning site in pFLUXH. Due to the use of NdeI site (CATATG), the native sequence of three nucleotides immediately upstream of the translational start codon was replaced with CAT and start codons were replaced with ATG. pFLUXH derivatives were introduced into M145 and P138 *Streptomyces strains* by conjugation. Negative control strain (MG01) was M145 with an empty pFLUXH plasmid. Two hundred microliter of solid modified 79 medium supplemented with higromycin (50 μg/ml) and 10 μM ZnCl_2_ if needed, was placed in wells of a white 96-well clear bottom plate and inoculated with 10 μl of spore suspension diluted in water to OD_600_ = 0.3 to give confluent growth. Cultures were incubated at 30°C directly in the ClarioStar microplate reader. Luminescence and OD_600_ were measured every hour.

## Results

### HypR Protein Binds Its Own Promoter Region

**FIGURE 1:**
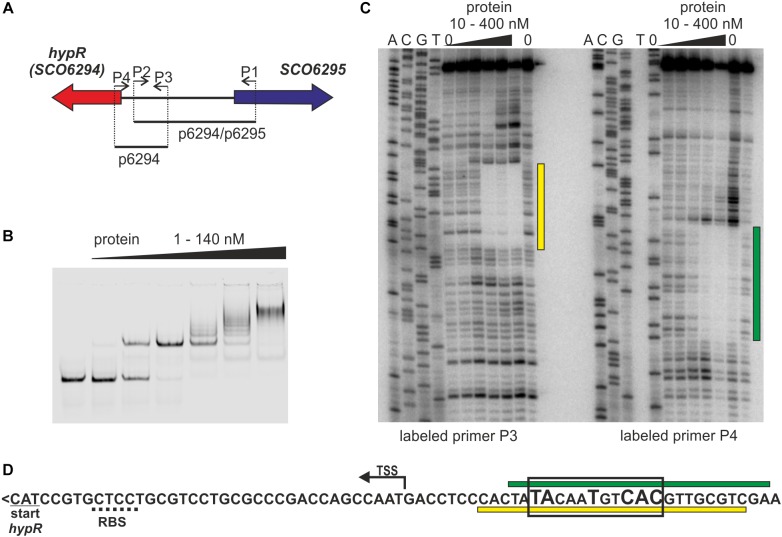
Identification of the DNA sequence recognized by HypR protein in its own promoter region. **(A)** Schematic representation of the location of primers and fragments used for EMSA and footprint experiments. **(B)** Binding of HypR protein to IR Dye 800 labeled fragment p6294/p6295 (EMSA). **(C)** Protection of the fragment p6294 from digestion by HypR protein (DNase I footprint). The radiolabeled primer is indicated below the lanes. Protected regions are marked with yellow and green bars. **(D)** Nucleotide sequence upstream of *hypR (SCO6294)* gene. Start codon and predicted ribosome binding site (RBS) are underlined with solid and dotted lines, respectively. TSS – transcription start site (Jeong et al., 2016). Protected regions from panel **(C)** are marked with yellow and green bars. Sequence similar to consensus motif identified in bacterial one hybrid system (see Supplementary Figure S2) is boxed and conserved nucleotides are bigger.

Looking for regulators of *cpk* gene cluster (genes *SCO6269 – SCO6288*) (Pawlik et al., 2007), coding coelimycin polyketide synthase (Pawlik et al., 2010; Gomez-Escribano et al., 2012), we started to investigate the role of a potential regulator coded by a neighboring gene *SCO6294* which we later named *hypR* (for L-hydroxyproline utilization regulator, as explained below). The protein was expressed in *E. coli* with N-terminal His-tag and Electrophoretic Mobility Shift Assay (EMSA) was used to test if it binds to promoters of *cpk* genes, but it was not the case (data not shown). Many GntR like proteins are autoregulators (Hoskisson and Rigali, 2009), therefore *hypR* promoter region was also included in the assay. Binding of the HypR protein was observed to the DNA fragment p6294/p6295 encompassing its own promoter region and that of the neighboring gene *SCO6295* (Figure 1A,B). EMSA experiments with two shorter fragments (not shown) indicated that the binding site is located immediately upstream of *hypR*.

The DNA sequence bound by HypR protein was found in the DNase I footprint experiment. The ^32^P labeled p6294 was incubated with increasing amount of His-tagged HypR protein and digested with DNase I (Figure 1C). The 21 bp sequence protected by HypR from DNase I digestion was found upstream of the *hypR* start codon covering the -10 region of the promoter (Figure 1D; see Supplementary Text S1 and Supplementary Figure S1 for new annotation of *hypR* start). No other similar sequences were found on *S. coelicolor* A3(2) chromosome.

To further characterize HypR binding sequence a bacterial one-hybrid system (B1H) was used (Meng et al., 2005). The studied DNA binding regulator was expressed as a fusion protein with the α-subunit of RNA polymerase in the selection strain *E. coli* US0 (Δ*hisB*Δ*pyrF*) (Meng et al., 2006) which is unable to grow on the minimal medium lacking histidine. The strain was transformed with a library of 18 bp randomized oligonucleotides cloned upstream of the reporter genes (the yeast HIS3 and URA3). Clones in which interaction between the fusion protein and the promoter region of reporter genes took place were identified by their ability to grow in the absence of histidine. Weak interactions were eliminated by the addition of 5 mM 3-AT (3-amino-triazole, a competitive inhibitor of HIS3). After screening the library, 35 individual clones were selected. Their randomized regions were sequenced and analyzed by a motif finding software MEME (Bailey and Elkan, 1994; Supplementary Figure S2). A consensus sequence 5′-TATNNTNNC(AC)(TC)-3′ recognized by HypR protein was found. A sequence similar to this motif (TACAATGTCAC) with one mismatch (underlined) is present within the region protected by bound HypR protein from digestion by DNase I (Figure 1D). The narrowed down sequence allowed us to identify other promoters recognized by HypR.

### HypR Protein Binds Zinc Ions

Predicted structure of HypR protein (details below) resembles that of TM0439 protein from *Thermotoga maritima* (Zheng et al., 2009), which represents a group of metal binding proteins from FadR subfamily of GntR-like regulators. The physiological role of TM0439 and its interaction with metal ions is unknown.

**FIGURE 2:**
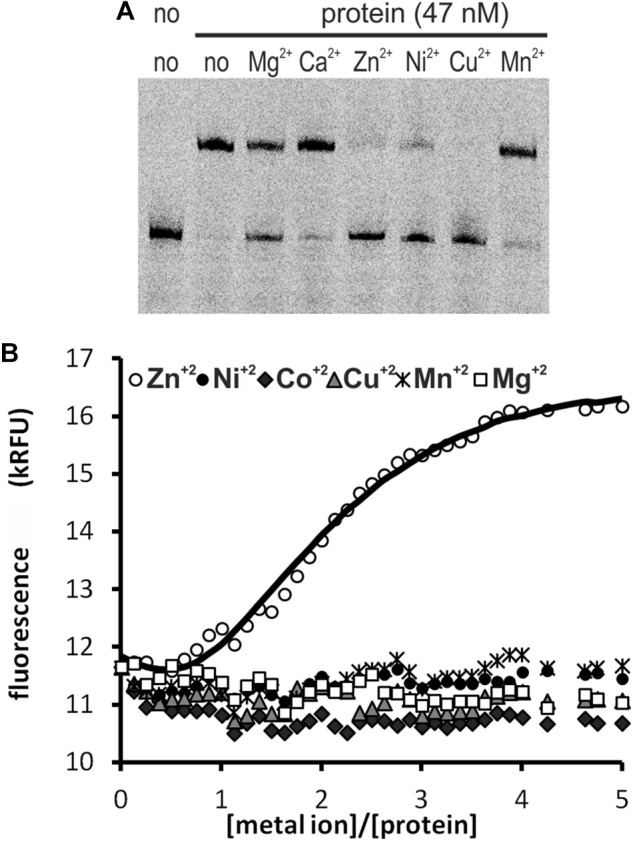
Interactions of HypR protein with metal ions. **(A)** Influence of divalent metal ions on binding of HypR protein to its own promoter region. Fragment p6294 was labeled with IR Dye 800, 5 mM metal ions were added to the samples, protein:DNA molar ratio was 25:1. **(B)** Tryptophan fluorescence of HypR protein in the presence of divalent metal ions. Solid line represents the best fit, found by DynaFit programme (Kuzmič, 1996) accounting for appropriate metal–buffer interactions (Zheng et al., 2009).

We tested the influence of several divalent metal ions on DNA binding by HypR. To avoid possible interactions of His-tag with the metal, we used in this experiment Strep-tagged HypR expressed in *E. coli* from pMS08 plasmid. As observed in EMSA analysis, DNA binding was abolished by the addition of divalent metal ions (Ni^2+^, Cu^2+^, and Zn^2+^) (Figure 2A). Magnesium ions had a weaker effect and the effect of calcium and manganese was negligible.

To assess binding of metal ions by HypR protein we measured its intrinsic tryptophan fluorescence. Samples containing Strep-tagged protein solution were titrated with zinc, nickel, cobalt, copper, manganese and magnesium chlorides. Zinc was the only metal which altered the intensity of fluorescence of the protein (Figure 2B). Zn^2+^ binding fitted the 2:1 (metal to protein) stoichiometric model with binding constants: K1 = (3 ± 1.5) × 10^7^ M^−1^, K2 = (6.5 ± 0.33) × 10^5^ M^−1^, comparable to those reported for TM0439 protein: K1 = (1.4 ± 0.1) × 10^7^ M^−1^, K2 = (4.5 _±_ 0.4) × 10^5^ M^−1^ (Zheng et al., 2009). Data fitting and model selection were done with DynaFit programme (Kuzmič, 1996). Obtained results indicate that zinc is the metal specifically bound by HypR.

### Identification of Genes Controlled by the HypR Protein

**FIGURE 3:**
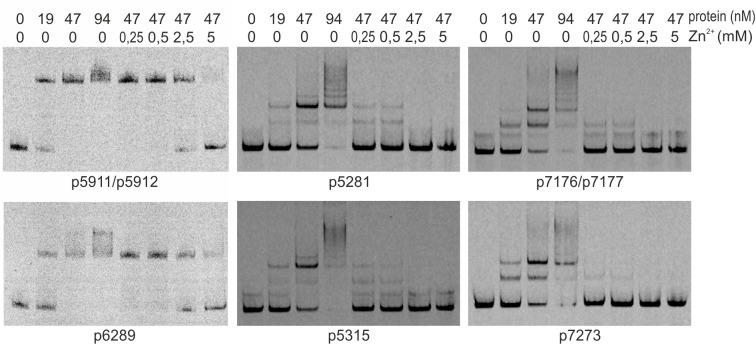
Binding of HypR protein to different promoter regions (EMSA). Fragments p5911/p5912 and p6289 were labeled with IRD-800 and incubated with Strep-tagged protein, remaining fragments were labeled with IRD-700. Protein and zinc chloride were added as indicated. Fragments for EMSA were between 104 and 129 bp long with the identified motifs (listed in Table 1) in the center.

**FIGURE 4:**
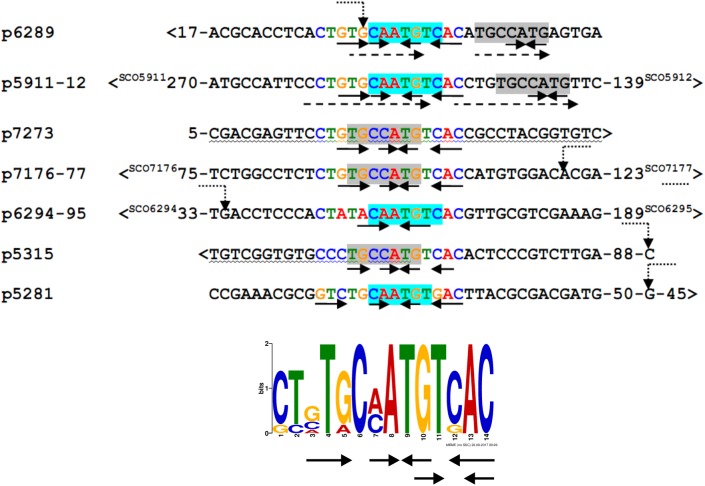
Alignment of DNA sequences of fragments bound by HypR protein and a 14 nt consensus sequence found by MEME program (*E*-value: 5.8e-012). Letters in the alignment are colored as in the consensus sequence logo. Gray or cyan background indicates identical sequences. Solid arrows – palindromic elements; dashed arrows – direct repeats with 1 mismatch; wavy underlining – coding sequence; >, <– gene direction; numbers – distance to start codons. If known, transcription start sites (TSS) are marked by bent dotted arrows (Jeong et al., 2016).

We searched for other sequences bound by HypR on *S. coelicolor* A3(2) chromosome with FUZZNUC program ^2^. We used the TACAATGTCAC sequence from *hypR* promoter allowing for one mismatch. Three locations in potential promoter regions were found (Table 1, positions 2–4). Two of them (promoter of *SCO6289* and intergenic region of *SCO5911* and *SCO5912*) were confirmed by EMSA to be recognized by HypR protein (Figure 3 and Supplementary Figure S3). In the second round of search the sequence TGCAATGTCAC common for both p6289 and p5911-12 fragments was taken, again allowing for one mismatch. Three sequences located in five putative promoter regions were found (Table 1, positions 5–9). Binding of four of them was confirmed by EMSA. Similarly as in case of *hypR* promoter, all the fragments were not bound in the presence of Zn^2+^ ions (Figure 3). DNA sequences of seven fragments bound by HypR protein were subjected to MEME analysis and a 14-nucleotide consensus sequence CTNTGC(A/C)ATGTCAC was found (Figure 4). Small palindromic elements and, in two cases, direct repeats can be noticed.

**Table 1:** Sequences similar to the motif from *hypR* promoter region found in potential promoter regions on *Streptomyces coelicolor* A3(2) chromosome.

No.	Sequence	SCO gene no.	Binding in EMSA	*hypR* deletion effect on promoter activity
1	TACAATGTCAC	*6294/6295*	Yes	*hypR* (*SCO6294*) – increase *SCO6295* – decrease
2	T**G**CAATGTCAC	*5911/5912*	Yes	*SCO5911* – not active, *SCO5912* – increase
3	T**G**CAATGTCAC	*6289* (predicted operon *6289-6293*)	Yes	Increase
4	TACAA**G**GTCAC	*3666*	No	Not tested
5	T**G**CAATGTCA**G**	*2476*	No	Not tested
6	T**G**CAATGT**G**AC	*5281*	Yes	Increase
7	T**G**C**C**ATGTCAC	*5315*	Yes	Not active
8	T**G**C**C**ATGTCAC	*7176/ 7177*	Yes	No change
9	T**G**C**C**ATGTCAC	*7273*	Yes	No change

*Mismatches are in bold. The sign “/” denotes location of the motif upstream of two divergent genes. Binding of the fragment by HypR protein in EMSA is indicated. Deletion effect – activity of promoter probe measured as luminescence in hypR deletion mutant P138 vs. wild type strain M145.*

### HypR Is a Repressor of *SCO6289-SCO6293* Genes

In order to study the physiological role of the regulator, *hypR* gene was deleted from *S. coelicolor* A3(2) chromosome (Supplementary Figure S4). The obtained mutant strain, *S. coelicolor* P138, was viable and we did not note differences in growth and colored metabolites production between the wild type M145 strain and P138 mutant.

**FIGURE 5:**
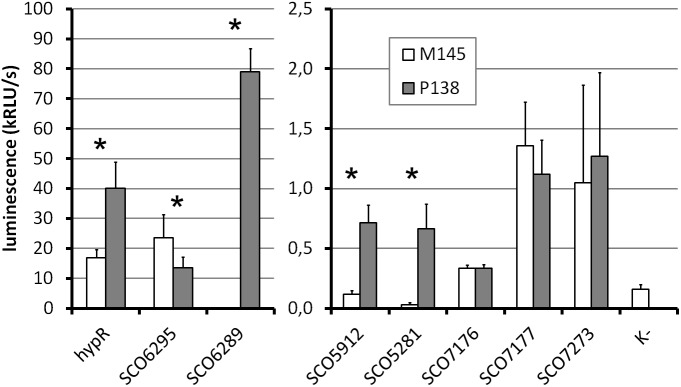
Promoter activity assay. Luminescence intensity after 45 h of growth of wild type (M145) and *hypR* deletion mutant (P138) strains carrying integrated promoter probe plasmids (pFLUXH derivatives). Negative control (K-) was M145 with an empty pFLUXH plasmid. Average values of data from at least three experiments are shown. Error bars represent standard deviation. Asterisks denote promoters with statistically significant differences of activity between M145 and P138 (*P* < 0.05 in *t*-test). The vertical axis description and legend refer to both parts of the graph.

To investigate the *in vivo* activity of promoters in the presence and absence of HypR protein, promoter probes based on pFLUXH vector (Supplementary Table S1) were introduced into M145 and P138 strains. The plasmid carries a promoterless luciferase operon luxCDAEB encoding luciferase and enzymes necessary for luciferase substrate (tetradecanal) biosynthesis (Craney et al., 2007). It allows direct monitoring of the luminescence in bacterial biomass. Luminescence of strains grown for 45 h is shown in Figure 5 (for time-course see Supplementary Figure S5). The same strains were grown on the medium supplemented with 10 μM ZnCl_2_ to check if the addition of zinc would have an effect on the activity of promoters *in vivo*, but the level of luminescence was not changed (Supplementary Figure S5).

The most striking differences between M145 and P138 were observed in case of *SCO6289*, *SCO5912*, and *SCO5281* promoters which showed activity only in P138. These results suggest that HypR is a repressor of probably co-transcribed genes *SCO6289-SCO6293* as well as *SCO5912* and *SCO5281*. Notably, promoter of *SCO6289* is much stronger than the remaining ones tested. Activity of *hypR* promoter was twice as high in P138 as in M145 strain. This indicates that HypR is an autorepressor. Wild type luminescence intensity in M145 was much higher for *hypR* promoter than for other identified target genes promoters, for which it was at the level of negative control, which means that the repression of its own promoter is weaker. The lower level of luciferase production from *SCO6295* promoter in the deletion strain (57% of that in wild type) suggests that the regulator may activate the expression of the transporter. Activity of *SCO7176*, *SCO7177*, and *SCO7273* promoters was not influenced by the deletion of *hypR* and promoters of *SCO5911* and *SCO5315* were not active in any of the strains.

### Annotation of Genes From HypR Regulon

**FIGURE 6:**
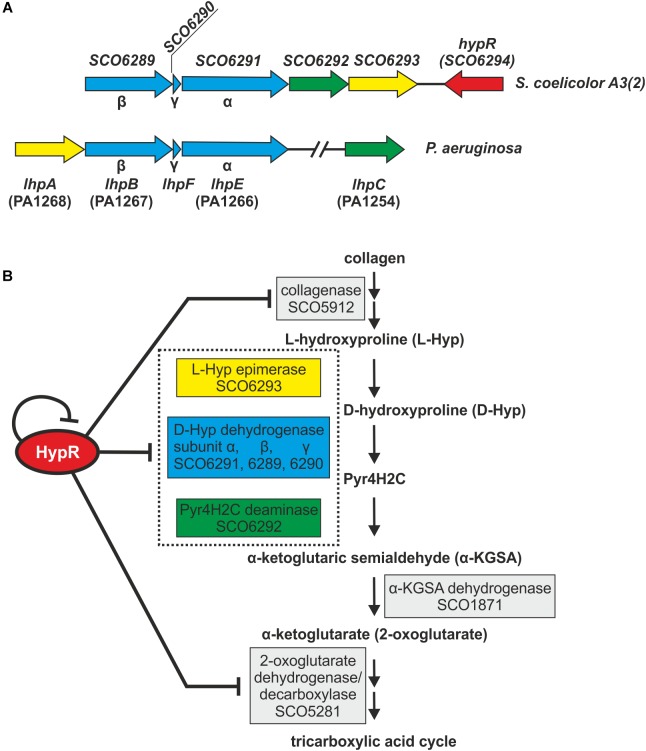
Schematic gene clusters for L-hydroxyproline metabolism in *Streptomyces coelicolor* A3(2) and *Pseudomonas aeruginosa* **(A)**. Interactions between HypR repressor and its regulatory target genes related to the predicted L-Hyp utilization pathway in *S. coelicolor* A3(2) (Watanabe et al., 2012; Chen et al., 2016) **(B)**. Pyr4H2C – Δ^1^-pyrroline-4-hydroxy-2-carboxylate, yellow – L-Hyp epimerase, blue – D-hydroxyproline dehydrogenase, green – Pyr4H2C deaminase. Dotted box represents enzymes coded in an operon. Influence of HypR protein on the activity of SCO1871 promoter was not tested.

Amino acid sequences and predicted structures of proteins coded by genes controlled by HypR transcription factor were subjected to similarity searches (Table 2). Genes *SCO6289-SCO6293* are probably co-transcribed, which indicates the involvement of the respective enzymes in a common pathway. The genes are conserved and share the same order on the chromosomes of *Streptomyces*, *Kitasatospora* and, to a lesser extent, *Pseudomonas* species, as shown by synteny analysis (Supplementary Figure S6; Oberto, 2013). They are predicted to code enzymes required for L-hydroxyproline (L-Hyp) utilization (Figure 6).

**Table 2:** Functions of the proteins from HypR regulon predicted on the basis of Blast (Altschul et al., 1990), Phyre2 (Kelley and Sternberg, 2009), and NCBI Conserved Domains search (Marchler-Bauer et al., 2017).

Protein	Amino acids	Predicted protein function	Conserved domains
HypR (SCO6294)	225	GntR-like regulator from FadR subfamily	GntR (COG1802)
SCO6295	662	ABC-type multidrug transport system, ATPase and permease component	MdlB (COG1132)
SCO6289	389	D-hydroxyproline dehydrogenase subunit β (secreted FAD dependent oxidoreductase, glycine/D-amino acid oxidase, zinc-containing alcohol dehydrogenases signature)	DAO (pfam01266), DadA (COG0665)
SCO6290	84	D-hydroxyproline dehydrogenase subunit γ (2Fe-2S iron-sulfur cluster binding protein)	Fer2_4 (pfam13510)
SCO6291	511	D-hydroxyproline dehydrogenase subunit α (oxidoreductase)	FadH2 (COG0446), soxA (TIGR01372), gltD (PRK12810), Fer2_BFD (pfam04324)
SCO6292	298	Δ^1^-pyrroline-4-hydroxy-2-carboxylate deaminase	DHDPS-like (cd00408), DapA (COG0329), dapA (TIGR00674)
SCO6293	333	L-hydroxyproline epimerase (proline racemase, 4-hydroxyproline epimerase)	Pro_racemase (pfam05544)
SCO5912	865	Class M9 secreted zinc metallopeptidase, collagenase	Peptidase_M9_N (pfam08453), Peptidase_M9 (pfam01752), PKD (pfam00801), PPC (pfam04151)
SCO5281	1272	2-oxoglutarate dercarboxylase, 2-oxoglutarate dehydrogenase	kgd (PRK12270)

L-Hyp is one of the main components of collagen. Its utilization by bacteria (Figure 6B) starts with conversion to D-hydroxyproline (D-Hyp) by L-Hyp epimerase, followed by oxidation to Δ^1^-pyrroline-4-hydroxy-2-carboxylate (Pyr4H2C) by D-Hyp dehydrogenase (D-HypDH), spontaneous hydrolysis and enzymatic deamination by Pyr4H2C deaminase to α-ketoglutaric semialdehyde (α-KGSA) and final conversion to α-ketoglutarate by α-KGSA dehydrogenase (Watanabe et al., 2012; Chen et al., 2016).

Conserved domains and molecular masses of proteins SCO6291, SCO6289, and SCO6290 correspond to α, β and γ subunits of D-HypDH from *Pseudomonas aeruginosa*, with amino acid sequence identity 34, 34, and 41% to respective proteins LhpE, LhpB, and LhpF (Watanabe et al., 2012; Figure 6A). SCO6293 and SCO6292 are homologs of L-Hyp epimerase and Pyr4H2C deaminase, respectively, with amino acid sequence identity 37 and 33% to respective proteins LhpA and LhpC from *P. aeruginosa*. L-Hyp catabolic pathway has also been described in *Sinorhizobium meliloti* (White et al., 2012). SCO6293 and SCO6292 have 44 and 32% identical amino acids, respectively, as *S. meliloti* L-Hyp epimerase (HypRE) and Pyr4H2C deaminase (HypD). However, the putative three subunit D-HypDH of *S. coelicolor* (SCO6289-91) has no significant homology with its functional counterpart in *S. meliloti* (HypO). D-HypDH enzymes may have different subunit compositions, while other enzymes of the pathway are conserved (Watanabe et al., 2012; Satomura et al., 2015).

Gene for α-KGSA dehydrogenase is not present in the proximity of the *SCO6289-SCO6293* operon, however, SCO1871 was found as a best hit (43–45% amino acid identity) when *S. coelicolor* A3(2) genome was searched for homologs of α-KGSA dehydrogenases of *P. aeruginosa*, *Pseudomonas putida*, *Azospirillum brasilense*, and *Sinorhizobium meliloti.*

The remaining genes repressed by the regulator include *SCO5912* coding a putative collagenase from class M9 metallopeptidases, as suggested by the order of conserved domains in SCO5912 protein and a presence of a zinc-binding motif HEXXH-E (Duarte et al., 2016), and *SCO5281* coding α-ketoglutarate (2-oxoglutarate) dehydrogenase or decarboxylase (Tian et al., 2005; Huergo and Dixon, 2015).

**FIGURE 7:**
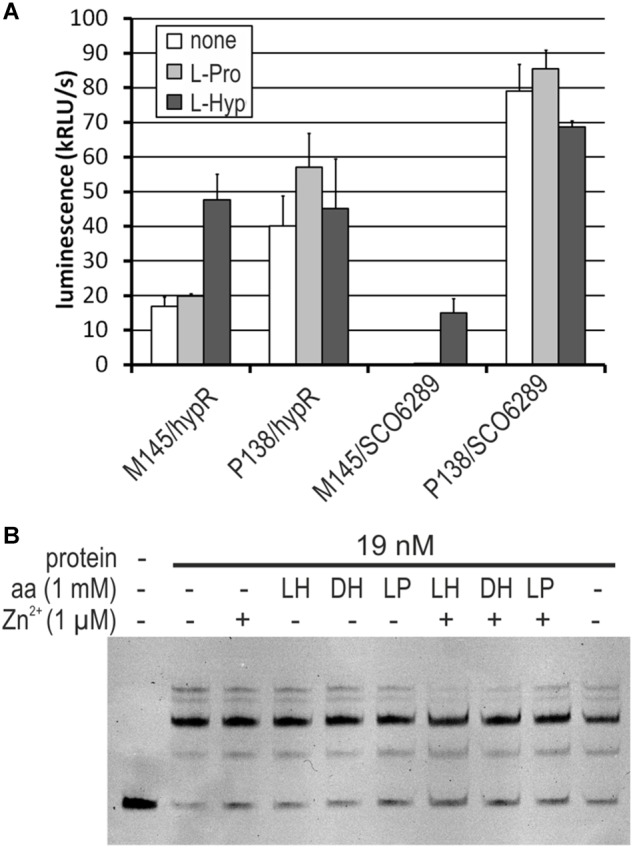
Influence of L-hydroxyproline (LH, L-Hyp), D-hydroxyproline (DH), and L-proline (LP, L-Pro) on the activity of HypR protein *in vitro* (DNA binding) and on the activity of promoters. **(A)** Activity of *hypR* and *SCO6289* promoters in M145 and P138 strains measured as luminescence after 45 h of growth on modified 79 medium with L-Hyp or L-Pro at the concentration 15 mM. **(B)** EMSA assay with IR Dye labeled fragment p6289. HypR protein, amino acids and zinc chloride were added as indicated.

We found that both M145 and P138 strains were able to grow in the liquid Minimal Medium (MM) with L-Hyp as a sole source of carbon. Growth testing on MM agar plates was unsuitable due to the ability of *S. coelicolor* A3(2) to utilize agar as a source of carbon. The luminescence reporter test showed that transcription of genes from the regulon of HypR was induced by L-Hyp in the wild type strain, while in the deletion mutant, promoter activity was independent from the amino acid addition (Figure 7A). Binding of DNA as tested by EMSA did not change in the presence of L-Hyp, D-Hyp, and L-proline with and without zinc ions (Figure 7B).

To verify if the operon SCO6289-SCO6293 is responsible for L-Hyp utilization, *SCO6293* gene, predicted to code L-Hyp epimerase was deleted. The obtained mutant strain *S. coelicolor* P201 lost the ability to grow in liquid MM with L-Hyp as a sole source of carbon, while it was still able to grow in the medium containing L-proline.

### HypR Protein Predicted Structure

HypR protein, as annotated in NCBI Protein Database under accession number NP_630392.1, has 263 amino acids. However, according to our results (see Supplementary Figure S1 and Supplementary Text S1) the annotation is based on an erroneous start codon and the actual protein is shorter – it consists of 225 aa and its calculated molecular mass is 24.6 kDa. In this work amino acids were numbered according to the corrected start. Similarity search using Blast (Altschul et al., 1990) revealed that HypR belongs to FadR subfamily of HTH GntR proteins and is highly conserved (28 hits with 80–94% identity covering 97–100% of the sequence). A model of three dimensional structure was obtained with Phyre2 program (Kelly et al., 2015) (confidence 100.0) based on the structure of TM0439 protein from *Thermotoga maritima* (Zheng et al., 2009) for which amino acid sequence identity and similarity are 29 and 53%, respectively (Supplementary Figures S7, S8). TM0439 is a dimer which is typical for FadR subfamily.

The winged helix-turn-helix (WHTH) N-terminal domain is formed by two helices α2 and α3 and two β strands. Based on comparison with crystal structures of FadR (Xu et al., 2001) and TM0439 (Zheng et al., 2009) the following amino acids of HypR are implicated in interactions with DNA: Ser50, Thr52, Arg55, Arg71, and Asn72 (Supplementary Figure S8).

C-terminal domains of GntR proteins from FadR subfamily are composed of six or seven α helices (Hoskisson and Rigali, 2009). Pfam database (Bateman et al., 2002) distinguishes a small FadR_C family (comprising FadR itself with seven α helices) (Pfam07840) and a large FCD family (Pfam007729) which includes C-terminal domains with both six and seven α helices. HypR protein has a six-helical ligand binding FCD domain. It has a potential metal ion binding site formed by three histidine residues His142, His186, and His208 which correspond to His134, His174, and His196 in TM0439 protein. In TM0439 two of the metal-binding histidines are stabilized by hydrogen bonds with Glu90, Asp130, and Glu173 (Zheng et al., 2009). Corresponding amino acids can be also found in HypR sequence (Glu96, Asp138, and Glu185). Mutational analysis of FCD domain of CitO protein, activator of citrate utilization pathway in *Enterococcus faecalis*, whose predicted structure is similar to HypR, revealed amino acids important for citrate binding and transcription activation *in vivo* (Arg97, His191, Phe143, Leu214) (Blancato et al., 2016). All the corresponding residues are conserved in HypR sequence (Arg92, His186, Phe141, Leu209) (Supplementary Figure S8).

## Discussion

### HypR Is a Repressor

Similarity search with amino acid sequence of HypR protein and analysis of its predicted structure shows that it belongs to FadR subfamily of GntR-like proteins. It has a typical N-terminal DNA binding WHTH domain and a metal binding C-terminal FCD domain composed of six α helices.

Recombinant protein HypR bound the DNA in the *hypR* promoter region as shown by EMSA and DNase I footprint experiments. The sequence protected from DNase I cleavage by binding of HypR to its own promoter corresponded to a motif identified in bacterial one-hybrid system (Figure 1). Binding of the protein was shown for six other DNA fragments with similar sequences located in potential promoter regions (Table 1 and Figure 3) and a consensus sequence motif CTNTGC(A/C)ATGTCAC shared by the fragments was found (Figure 4). Finally, HypR protein was shown by the luciferase reporter assay to be a repressor of *SCO6289-SCO6293* operon, *SCO5912*, and *SCO5281* genes and an autorepressor (Figure 5). The repression of its own promoter is weaker than that of other target genes.

Most of the studied GntR-like proteins bind as dimers to twofold symmetric DNA operator sequences, with each monomer recognizing a half-site (Suvorova et al., 2015). Oligomeric state of HypR was not studied. We expect it to be a dimer based on the predicted structure similarity to the dimeric protein TM0439. Symmetry of the consensus binding site sequence of HypR protein is not clear, but short palindromic elements can be noticed (Figure 4) including the GT/AC pair resembling the central part of the conserved motif TKGT/ACMA found for FadR subfamily (Suvorova et al., 2015).

The HypR binding DNA consensus sequence motif is located in diverse positions relative to transcription start sites (TSSs) of respective genes (Figure 4). In case of FadR, it is the exact location of its recognized sequence that determines whether the bound regulator represses or activates target genes (Xu et al., 2001). The binding site of HypR in its own promoter covers the -10 region, which is consistent with its role as a repressor. Similarly, binding of the repressor next to the TSS of *SCO6289* should impair access of RNA polymerase. On the other hand, the consensus sequence motif is located more than 60 bp upstream of TSS of *SCO5281*, a location resembling that of an activator CitO, which binds upstream of -35 regions (Blancato et al., 2008). Observed decrease of activity of *SCO6295* promoter in *hypR* deletion strain suggests that the regulator may activate the expression of the transporter. However, HypR binding site in its own promoter is located as much as 200 bp upstream of the start codon of the divergent gene *SCO6295*, therefore we consider an indirect effect more likely than direct activation.

### Zinc Ion Is a Cofactor of HypR

Majority of FCD domains have conserved histidine residues for coordinating a divalent metal ion (Zheng et al., 2009). Unlike in metal-sensing transcription factors regulating metal uptake/efflux systems, which have metal binding sites at or near dimer interface (Giedroc and Arunkumar, 2007), in FCD domains the site is buried at the bottom of a cavity formed by α helices. The size of the cavity is suitable to accommodate a small organic compound (Zheng et al., 2009). Although it is not known whether acetate presence in the FCD domain of TM0439 is physiologically relevant, its location in the crystal structure helped to identify amino acids important for formation of the ligand binding pocket in CitO from *Enterococcus faecalis* (Blancato et al., 2016). The same amino acids can be found in respective positions in HypR (Supplementary Figure S8).

CitO activates citrate utilization pathway by binding *cit* promoters in response to sensing citrate (Blancato et al., 2008). It was shown, that coordinating the metal ion is crucial for binding citrate molecule and for effective DNA binding. The authors proposed that the ligand interacts with the buried Ni^2+^ or Zn^2+^ ion in the FCD domain leading to conformational changes of the N-terminal domain that improve recognition of DNA target. DNA binding by CitO-citrate complex *in vitro* is not disturbed by the addition of metal ions. In the absence of citrate, binding of DNA by the recombinant CitO is much weaker and sensitive to nickel and zinc (Blancato et al., 2016).

Similarly, DNA binding by HypR protein was prevented by several divalent metal ions (Figure 2A). However, changes of tryptophan fluorescence were observed only in case of zinc (Figure 2B). If zinc ion had a signaling function and its binding caused dissociation of the protein from its target sequences in the promoter regions, transcription of respective genes should increase in its presence. However, we found that the addition of ZnCl_2_ to the medium did not change promoter activity (Supplementary Figure S5). We suspect that zinc ion is a cofactor involved in binding of an organic compound in a way similar to citrate binding by CitO.

### Predicted L-Hydroxyproline Utilization Pathway

None of the proteins from HypR regulon have been previously studied experimentally. On the basis of sequence and predicted structure similarity searches (Table 2) we propose that they are involved in the degradation of collagen, metabolism of one of its components (L-hydroxyproline) and directing the product (α-ketoglutarate) to the TCA cycle for production of energy and biosynthetic precursors (Figure 6). We have found here that deletion of putative L-Hyp epimerase gene (*SCO6293*) leads to the loss of the ability of *S. coelicolor* A3(2) to grow in the minimal medium containing L-Hyp as a sole source of carbon. This confirms the involvement of *SCO689-93* genes in L-Hyp catabolism. Transcription of the operon *SCO689-93* and *hypR* gene is induced by L-Hyp in wild type strain, and not in *hypR* deletion mutant (Figure 7). Interestingly, *SCO6289* promoter activity reached approximately 20% of the value obtained in the absence of the repressor. However, neither L-Hyp nor D-Hyp had any effect on DNA binding by HypR *in vitro*. This suggests a different effector compound interacting with the repressor (likely a downstream metabolite). It is also possible that transcription induction by L-Hyp does not lead to HypR protein dissociation from the DNA but to a conformational change and/or involves additional factors. Known L-Hyp uptake and utilization pathways of other bacteria are induced by both L-Hyp and D-Hyp, but the molecular mechanisms of transcription regulation are different. In *Pseudomonas aeruginosa* the expression of *lhp* genes is activated by LhpR protein of the AraC family as a result of binding L-Hyp (Li et al., 2016). In S*inorhizobium meliloti* the *hyp* genes are controlled by a GntR (FadR)-like repressor HypR. We chose the same name for SCO6294 protein. Analysis of transcription induction in a series of deletion mutants indicates D-Hyp (or a downstream metabolite) as the most likely effector molecule interacting with *S. meliloti* HypR, but DNA binding tests were not performed (White et al., 2012).

L-Hyp is one of the main components of collagen. It is also present in plant and algae cell walls and in root nodules. Therefore, it is an abundant carbon and nitrogen source for soil-dwelling bacteria (Moe, 2013). However, the ability to hydrolyse collagen and to metabolize L-Hyp is not a common feature of bacteria. It is utilized by some pathogens, such as Pseudomonads, which degrade collagen as a part of invasion of host tissues (Duarte et al., 2016). Molecular mechanism of L-Hyp utilization was also described for nitrogen-fixing plant endosymbiont *S. meliloti* (White et al., 2012). To our knowledge, the current work is the first indication of the existence of the same pathway of L-Hyp metabolism in Streptomycetes, although collagenases from this genus were reported previously (Sakurai et al., 2009) and gene clusters homologous to SCO6289-93 can be found in many *Streptomyces* genomes (Supplementary Figure S6). It gives a new insight into the way of adaptation of the model organism *S. coelicolor* A3(2) to nutrients from plant and animal tissues available in the soil. Moreover, enzymes from HypR regulon may find biotechnological applications. Collagenases are used, among others, in food technology and medical industry (Duarte et al., 2016). Putative L-Hyp dehydrogenase from *S. coelicolor* A3(2) belongs to Dye-linked D-amino acid dehydrogenases which can be applied to construct electrochemical biosensors (Satomura et al., 2015).

## Author Contributions

MK and KP designed the study and evaluated results. MK, MŚ, JZ-P, and PJ conducted the experiments. MK wrote the manuscript.

## Conflict of Interest Statement

The authors declare that the research was conducted in the absence of any commercial or financial relationships that could be construed as a potential conflict of interest.

## Footnotes

1 http://meme-suite.org/

2 www.hpa-bioinfotools.org.uk/pise/fuzznuc.html
