# Supplementary material for: A GntR-Like Transcription Factor HypR Regulates Expression of Genes Associated With L-Hydroxyproline Utilization in Streptomyces coelicolor A3(2)
Source: Front Microbiol. 2019 Jun 26;10:1451. doi: 10.3389/fmicb.2019.01451 (PMC6608401; doi:10.3389/fmicb.2019.01451)
Supplement: Supplementary file 1 [file Data_Sheet_1.pdf]

Supplementary Material

TABLE S1 Oligonucleotides used in this work. Restriction sites are in bold.

**Name**	**Sequence 5'-3'**	**Restriction sites**	**Application/amplified fragment**
**bacterial one-hybrid system**			
B1	CA**GGTACCGCGGCCGC**GATGCGCGCCGTCGCCGCGGGAC	KpnI, NotI	*hypR (SCO6294)* gene
B2	AT**GGATCCTAGG**TCAGTCCTCGGCGTTCGTCCTG	BamHI, AvrII	
B3	ATAAACTGCCAGGCATCAAATTAA		sequencing
B4	CGCTCGGTCGACACAACATACGA		sequencing
***hypR (SCO6294)* deletion**			
D1	**CTGCAG**TGAACTGACGATGCGTTCG	PstI	
D2	**GGATCC**GACTGAGCCGAAGAGCAC	BamHI	
D3	**GGATCC**ACGCAGGTCACCGGCGAT	BamHI	
D4	**GAATTC**GATCAAGCAGCAACTCGGT	EcoRI	
D5	**GGATCC**ACCAGCCAATGACCTCCC	BamHI	
D6	ATCAAGCTCGGCCAGGAG		*hypR* deletion confirmation
D7	AAGGTCACCGCCTCGAAG		*hypR* deletion confirmation
D8	CTCCCGCCGTCATCAAGT		*hypR* deletion confirmation
***SCO6293* deletion**			
D9	AAA**AAGCTT**CGCCTACGACCGTCAACT	HindIII	
D10	AAA**GCGGCCGC**TCGAACATCGTG	NotI	
D11	AAA**GCGGCCGC**TTTCACCCTCTGAGCAC	NotI	
D12	AAA**GGTACC**GCCGGTAGATCTGGTTG	KpnI	
D13	GTTCGACGGTCTGCTACCAC		*SCO6293* deletion confirmation
D14	AAGTACCTCGAAGCCGATCA		*SCO6293* deletion confirmation
***hypR (SCO6294)* gene cloning**			
G1	**GGATCCCATATG**CGCGCCGTCGCCGCGGG	BamHI, NdeI	*hypR (SCO6294)* gene
G2	**GAGCTCAAGCTTCTAGA**TCAGTCCTCGGCGTTCGTCC	SacI, HindIII, XbaI	
**fragments for EMSA assay**			
P1	TGTAGCGGATGCCGATGTT		p6294/p6295
P2	CAGCCAATGACCTCCCACTA		
P3	TTCCACGAGGCCGCGATG		p6294
P4	CATCCGTGCTCCTGCGTCCT		
p5911-12_F	ACGGTCTCACTCCTCGATG		p5911/p5912
p5911-12_R	GACGGAAGACGAACGACCT		

p6289_F	ACGACGACCTTCAGGGTCAC		p6289
p6289_R	GTGAAGCGGGCGTTGTCT		
p3666_F	CGTGCTGCCTACTTCAGACC		p3666
p3666_R	AGGCCAACATCGAGGAGAC		
p2476_F	GGTCCCTTTTCGCAGACTTT		p2476
p2476_R	GCTGGGTGACCGGGACCA		
p5281_F	CTCACGCGGCAGAACCTC		p5281
p5281_R	GGAATCAAGGCTACGCCTAC		
p7176-77_F	ATCGATCGAAGTCAGCCATC		p7176/p7177
p7176-77_R	ACACGGTGCAGTGTGTCG		
p7273_F	GCAGGCGGGCTGAGTGAC		p7273
p7273_R	GTGGGGGCCCGGTAGGTG		
**fragments for luciferase assay (promoter probes)**			
P5	**GGATCC**CACCGTCACACTCCCGGA	BamHI	p6294-old – fragment incorrectly indicated as a potential promoter region of *hypR (SCO6294)* gene due to misannotation
P6	**CATATG**GGCCTCGTGGAAGGCCCG	NdeI	
P7	**GGATCC**CCGCGATGCGCGCCGTCG	BamHI	p6294-probe – promoter region of *hypR* gene
P8	**CATATG**TGCTCCTGCGTCCTGCGC	NdeI	
p5281_Bam	**GGATCC**TGACGTAGGGTTCGAAGC	BamHI	p5281-probe
p5281_Nde	**CATATG**GAGAACCGCCCTCCTTCC	NdeI	
p5315_Bam	**GGATCC**TGTCGGCGCTGATGAAG	BamHI	p5315-probe
p5315_Kpn	**GGTACC**GATCTCGTTGTCGGTGTG	KpnI	
p5911-12_Nde_F	**CATATG**GCTCGTCCTCGCAAGATC	NdeI	p5911/p5912-probe
p5911-12_Nde_R	**CATATG**GCCTCCCATGAGGGATCC	NdeI	
p6295_Bam_F	**GGATCC**CATCCGTGCTCCTGCGTC	BamHI	p6295-probe
p6295_Nde_R	**CATATG**CACACTCCCGGAGGTTCG	NdeI	
p6289_Bam_F	**GGATCC**TGACGCGCCCGATCTCGA	BamHI	p6289-probe
p6289_Nde_R	**CATATG**CTCCTCCTCCGTGCACG	NdeI	
p7176-77_Nde_F	**CATATG**GACCTCACCGACTAGATG	NdeI,	p7176/p7177-probe
p7176-77_Nde_R	**CATATG**TCTCCCACCCTGACCCCA	NdeI,	
p7273_Bam	**GGATCC**ATGGCGATCAGCCTCCA	BamHI	p7273-probe
p7273_Kpn	**GGTACC**GACCGTCACAGACACC	KpnI	
**other**			
P9	GCTCTCGGGGAAGATCTCGAC		control of cloning in pFLUXH
P10	IRDye-ATGCAGGCCTCTGCA		amplification and IRD labeling of fragments cloned in pTZ57R
P11	IRDye-TCGGTACCTCGCGAA		
P12	**TAGCTA**CTGCGCCTCCACAAG		*SCO6281* promoter, non-specific competitor for EMSA
P13	CGACGGCACCGTGTCTGATGA		

TABLE S2 Bacterial strains and plasmids used in this study

**Strain or plasmid**	**Relevant genotype or description**	**Source or reference**
Escherichia coli		
DH5α	F^- endA1 glnV44 thi-1 recA1 relA1 gyrA96 deoR nupG Φ80dlacZΔM15 Δ(lacZYA-argF)U169, hsdR17(r_K^- m_K^+), λ^-	Promega
BL21(DE3)	F-, *ompT*, *hsdSB* (rB-, mB-), *gal*, *dcm* (DE3)	Promega
BL21(DE3)pLysS	F-, *ompT*, *hsdSB* (rB-, mB-), *gal*, *dcm* (DE3), pLysS (Cam^R)	Promega
US0(ΔhisBΔpyrF)	selection strain for bacterial one-hybrid system with deletion in *hisB* and *pyrF* genes (homologues of yeast *HIS3* and *URA3*)	(Meng et al., 2006)
ET12567/pUZ8002	strain for conjugal transfer of DNA from *E. coli* to *Streptomyces* (*dam dcm hsdS* Cam^R Tet^R on the bacterial chromosome; *tra* Kan^R RP4 23 on pUZ8002)	(Kieser et al., 2000)
***Streptomyces coelicolor* A3(2)**		
M145	wild type strain, *S. coelicolor* A3(2) ($SCP1^-$ $SCP2^-$)	(Kieser et al., 2000)
P130	deletion of *hypR (SCO6294)* gene (replacement with hygromycin resistance cassette Ω*hyg*)	this work
P138	deletion of *hypR (SCO6294)* gene (no resistance cassette)	this work
P201	deletion of *SCO6293* gene	this work
MG01	negative control for luciferase assay, M145 with integrated pFLUXH without insert	(Szafran et al., 2016)
strains for luciferase assay	M145 and P138 with integrated pFLUXH derivatives for luciferase assays	this work
**Plasmids**		
pGEM-T Easy	T-vector for direct cloning of PCR products (Amp^R)	Promega
pTZ57R/T	T-vector from InstT/A Cloning kit for direct cloning of PCR products (Amp^R)	Fermentas (Thermo-Scientific)
pET28a(+)	plasmid for expression of proteins with His-tag (T7 promoter, Kan^R)	Novagen
pET28NStrep	pET28a(+) vector modified by insertion of the sequence generating N-terminal Strep-tag between NcoI and BamHI sites	(Jaworski et al., 2018)
pH3U3-18random	the library of randomized 18-bp sequences upstream of reporter genes (the yeast *HIS3* and *URA3*) in the pH3U3 plasmid (Kan^R)	(Wolański et al., 2011)
pB1H1	plazmid (Cam^R) for expression of proteins fused to α subunit of RNA polymerase	(Meng et al., 2005)
pB1H1-SCO6294	plazmid pB1H1 with *hypR (SCO6294)* gene cloned in NotI and BamHI sites providing fusion of HypR with α subunit of RNA polymerase	this work
pOJ260	vector for conjugal transfer from *E. coli* to *Streptomyces*, non-integrative, ($Apra^R$), does not propagate in *Streptomyces*	(Bierman et al., 1992)
pIJ2529-hyg	pIJ2529 (Kieser et al., 2000) with hygromycin resistance cassette Ω*hyg* (Blondelet-Rouault et al., 1997) cloned in BamHI site	gift from A. Butler, University of Leicester, UK
pJZ12	pOJ260 with *upstream* flanking arm amplified using the primer pair D3 and D4 and cloned in BamHI and EcoRI sites	this work
pJZ13	pJZ12 with *downstream* flanking arm amplified using the primer pair D1 and D2 and cloned into BamHI and PstI sites	this work

pJZ14	pJZ13 containing hygromycin resistance cassette Ω*hyg* obtained as BamHI restriction fragment from pIJ2925-hyg plasmid and cloned in BamHI site of pJZ13 between *upstream* and *downstream* flanking arms	this work
pJZ15	pJZ12 in which *upstream* flanking arm was replaced by *upstream2* flanking arm amplified using the primer pair D4 and D5 and cloned into BamHI and PstI sites	this work
pGR1	*SCO6294* gene amplified with G1 and G2 primers cloned in pGEM-T Easy vector	this work
pJZE1	expression plasmid pET28a(+) with *hypR (SCO6294)* gene cloned in NdeI and HindIII sites to generate His-tagged HypR protein	this work
pMS08	expression plasmid pET28NStrep with *hypR (SCO6294)* gene cloned in BamHI and HindIII sites to generate Strep-tagged HypR protein	this work
pIJ12738	vector for conjugal transfer from *E. coli* to *Streptomyces*, non-integrative, ($Apra^R$), does not propagate in *Streptomyces*, containing the I-SceI recognition sequence	(Fernandez-Martinez and Bibb, 2014)
pIJ12742	temperature sensitive I-SceI meganuclease delivery vactor, ($Apra^R$, Thi^R)	(Fernandez-Martinez and Bibb, 2014)
pMK43	pIJ12738 with right flanking arm amplified using the primer pair D11 and D12 and cloned in KpnI and NotI sites	this work
pMK44	pMK43 with left flanking arm amplified using the primer pair D9 and D10 and cloned in HindIII and NotI sites	this work
pFLUXH	ΦBT1 integrating reporter plasmid with a promoterless luciferase operon *luxCDAEB*	(Craney et al., 2007) modified by Szafran *et al.* (Szafran et al., 2016)
pFLUXH derivatives	pFLUXH with promoter fragments for luciferase assay listed in table S1 in the supplementary material	this work

Bierman, M., Logan, R., O'Brien, K., Seno, E. T., Nagaraja Rao, R., and Schoner, B. E. (1992). Plasmid cloning vectors for the conjugal transfer of DNA from Escherichia coli to Streptomyces spp. *Gene* 116, 43–49. doi:10.1016/0378-1119(92)90627-2.

Blondelet-Rouault, M. H., Weiser, J., Lebrihi, A., Branny, P., and Pernodet, J. L. (1997). Antibiotic resistance gene cassettes derived from the Ω interposon for use in E. coli and Streptomyces. *Gene* 190, 315–317. doi:10.1016/S0378-1119(97)00014-0.

Craney, A., Hohenauer, T., Xu, Y., Navani, N. K., Li, Y., and Nodwell, J. (2007). A synthetic luxCDABE gene cluster optimized for expression in high-GC bacteria. *Nucleic Acids Res.* 35, 1–10. doi:10.1093/nar/gkm086.

Fernández-Martínez, L. T., Bibb, M. J. (2014) Use of the meganuclease I-SceI of Saccharomyces cerevisiae to select for gene deletions in actinomycetes. Sci Rep. 4, 7100. doi:10.1038/srep07100.

Jaworski, P., Donczew, R., Mielke, T., Weigel, C., Stingl, K., and Zawilak-Pawlik, A. (2018). Structure and Function of the Campylobacter jejuni Chromosome Replication Origin. *Front. Microbiol.* 9, 1–18. doi:10.3389/fmicb.2018.01533.

Kieser, T., Bibb, M. J., Buttner, M. J., Chater, K. F., and Hopwood, D. A. (2000). Practical Streptomyces Genetics. *John Innes Cent. Ltd.*, 529. doi:10.4016/28481.01.

Meng, X., Brodsky, M. H., and Wolfe, S. A. (2005). A bacterial one-hybrid system for determining the DNA-binding specificity of transcription factors. *Nat. Biotechnol.* 23, 988–994. doi:10.1038/nbt1120.

Meng, X., Smith, R. M., Giesecke, A. V., Joung, J. K., and Wolfe, S. A. (2006). Counter-selectable marker for bacterial-based interaction trap systems. *Biotechniques* 40, 179–184. doi:10.2144/000112049.

Szafran, M. J., Gongerowska, M., Gutkowski, P., Zakrzewska-Czerwińska, J., and Jakimowicz, D. (2016). The coordinated positive regulation of topoisomerase genes maintains topological homeostasis in Streptomyces

coelicolor. *J. Bacteriol.* 198, 3016–3028. doi:10.1128/JB.00530-16.

Wolański, M., Donczew, R., Kois-Ostrowska, A., Masiewicz, P., Jakimowicz, D., and Zakrzewska-Czerwińska, J. (2011). The level of AdpA directly affects expression of developmental genes in Streptomyces coelicolor. *J. Bacteriol.* 193, 6358–6365. doi:10.1128/JB.05734-11.

TEXT S1 Verification of *hypR* (*SCO6294)* gene annotation.

Alignments of HypR (SCO6294) sequence deposited in NCBI Protein Database (NP_630392.1) with 100 highest scoring Blast hits showed that significant similarity started from Met39 which corresponded to the N-terminal methionines of other proteins. N-terminal sequence of 38 amino acids showed similarity only to a corresponding protein (EFD65697.1) from *Streptomyces lividans* TK24, a very close relative of *S. coelicolor* A3(2). However, in another sequencing project the same protein from *S. lividans* TK24 is annotated without the initial 38 amino acids (AIJ12350.1). It is an indication, that the *SCO6294* gene annotation may be incorrect, and that the ATG codon corresponding to Met39 may be the real translational start. There is a potential ribosome binding site upstream of the corrected start codon. Moreover, it is consistent with the location of transcription start site of *SCO6294* gene detected by Jeong et al. (Nat Commun 7:11605, 2016, doi: 10.1038/ncomms11605) (see Fig. S1 A). In order to confirm this hypothesis, two fragments covering potential promoter of *hypR* gene, p6294-old and p6294-probe (**Fig. S1 A**), were cloned into pFLUXH plasmid upstream of reporter genes. The plasmid carries a promoterless luciferase operon *luxCDAEB* encoding luciferase and enzymes necessary for luciferase substrate (tetradecanal) biosynthesis (Craney *et al*, Nucleic Acids Res 35(6):e46, 2007). It allows direct monitoring of the luminescence in bacterial biomass. Each of the two promoter probe plasmids was introduced into *S. coelicolor* M145 strain by conjugation. High luciferase activity was observed only for fragment p6294-probe. No luminescence was detected for p6294-old fragment (**Fig. S1 B**). This leads us to the conclusion that the active promoter is located immediately upstream of the corrected start codon and that HypR protein consists of 225 amino acids (without the 38 N-terminal amino acids present in the NP_630392.1 sequence).

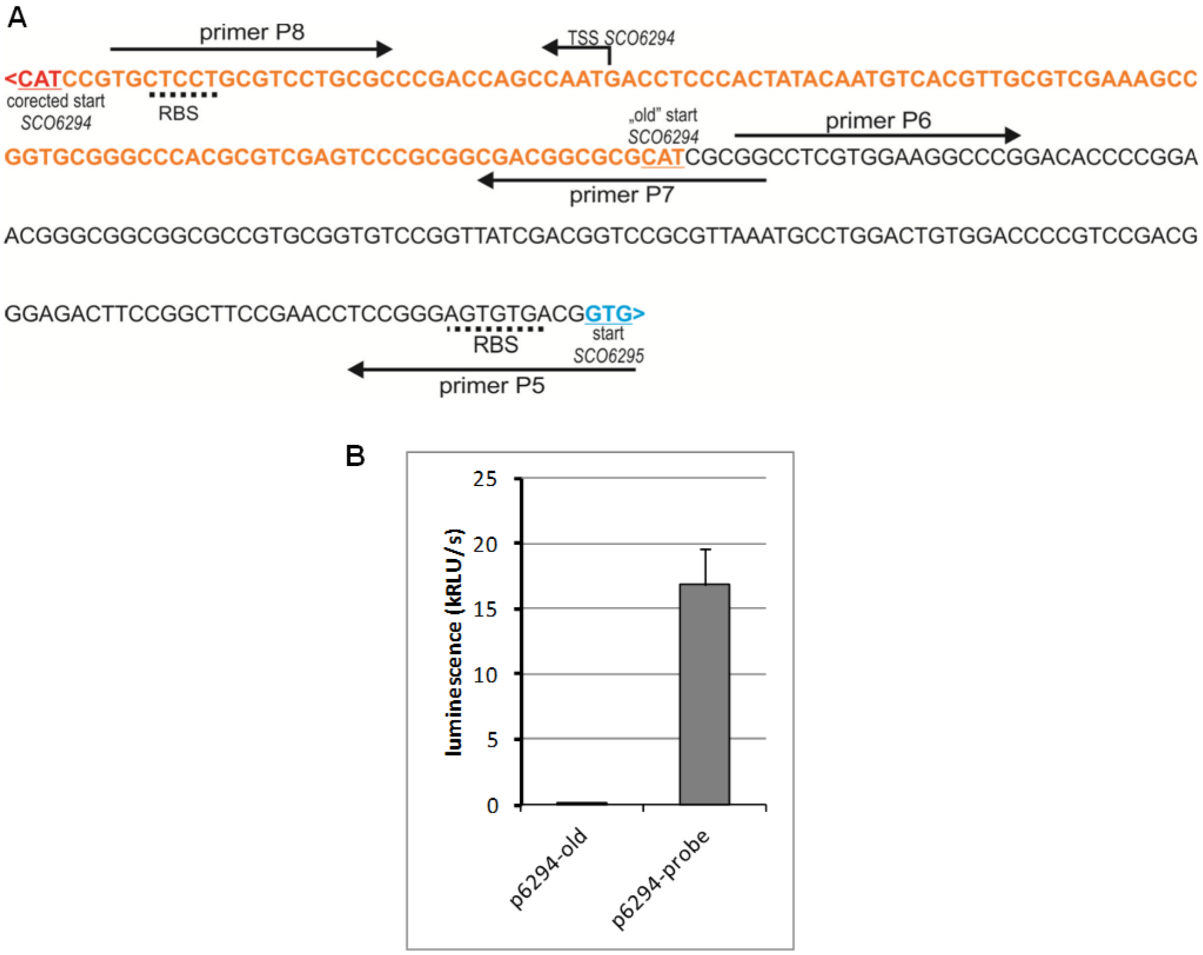

FIGURE S1 Verification of *hypR (SCO6294)* annotation. **(A)** DNA sequence of the fragment between *hypR* and *SCO6295* genes (respective start codons are marked with red and blue). Orange – fragment misannotated in EMBL/GenBank (Accession No. AL645882) as coding 38 N-terminal amino acids of SCO6294 (protein ID NP_630392.1). Start codons are underlined with solid lines and predicted ribosome binding sites (RBS) are underlined with dotted lines. TSS – transcription start site of *SCO6294* (Jeong *et al*, Nat Commun 7:11605, 2016, doi: 10.1038/ncomms11605). Location of primers is shown with arrows above the text (if the primer 5’->3’ sequence is in the same orientation) and below the text (in case of opposite orientation). Primer pairs P5, P6 and P7, P8 (see **Table S1**) were used to amplify DNA fragments p6294-old and p6294-probe located above the “old” (misannotated) and corrected start sites, respectively. **(B)** Comparison of luminescence intensity after 45 h of growth of *S. coelicolor* M145 strains carrying integrated promoter probe plasmids (pFLUXH derivatives) with either p6294-old or p6294 fragment. Average values of data from three experiments are shown. Error bars represent standard deviation.

Name	Sequence		
KNRA68	T	TATGCTATCAT	TCCTAC
KNRA61	T	TATGCTATCAT	TCCTAC
KNRA25	T	TATGCTATCAT	TCCTAC
KNRA81	AGCTCTC	TATGATACCGC	
KNRA64	TTTAACT	TAGGATACCAC	
KNRA48	AGCTCTC	TATGATACCGC	
KNRA77	TGC	TATGATACAAT	TAGT
KNRA73	G	TATGCTACACT	GCGACT
KNRA71	G	TATGCTACACT	GCGACT
KNRA76	G	TATCCTGTCAT	AATGCG
KNRA56	G	TATCCTGTCAT	AATGCG
KNRA79		TCTGGTACCGC	AAATGAA
KNRA78	AGTGCA	TAAGCTTACAC	G
KNRA59	AGTGCA	TAAGCTTACAC	G
KNRA80		TCTGATTACCT	TTGAGTC
KNRA66		TCTGATTACCT	TTGAGTC
KNRA58	GC	TAACATACTAT	AGCGA
KNRA54	GC	TAACATACTAT	AGCGA
KNRA69	AATAT	TAGTAAAACCC	CC
KNRA62	GGGAT	TCTTCAGCCCC	TG
KNRA55	GGGAT	TCTTCAGCCCC	TG
KNRA63	TCCC	TAATCTCAAAT	AAA
KNRA37	CGTATA	TATGGAGCGAT	C
KNRA75	CCG	GAGCCTCACCT	GCCT
KNRA74	CCG	GAGCCTCACCT	GCCT
KNRA72	CCCTACC	GATTGTAATAC	
KNRA21	CGTGTGT	TCGTGTTACAC	
KNRA45	GTGC	TATTATGTTGC	CGG
KNRA57		TAGTGTGCGAT	TAAGGGC
KNRA53	ACATGGG	TATTATATCGA	
KNRA65	TGA	TATAATCAACC	AGCG
KNRA60	TGGACCC	CATGGTAGCAT	
KNRA52	AATCGTC	GCTGTAAACAT	
KNRA70	TT	TATGTCCCAAC	TCCTT
KNRA67	TCTTTT	AATGAACATAC	C

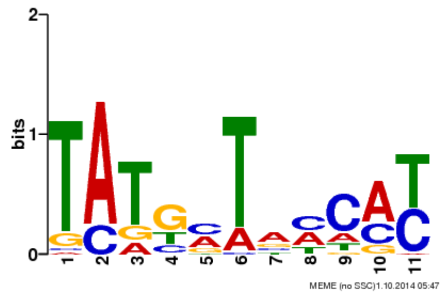

FIGURE S2 Sequences from 35 clones identified in bacterial one hybrid system as those in which interaction between the HypR protein and the promoter region of reporter genes took place and the consensus sequence logo (E-value: 1.5e-10) derived from them by the MEME algorithm.

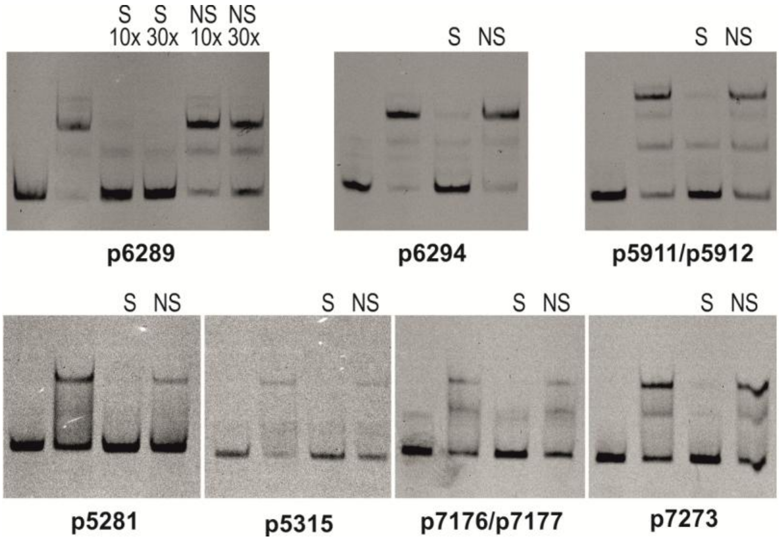

FIGURE S3 Binding of HypR protein to IR Dye 800 labeled promoter fragments indicated below each panel. First lane in each panel contains no protein. HypR concentration in the remaining lanes was 19 nM. S – specific competitor (unlabeled probe), NS – non-specific competitor (unlabeled promoter of *SCO6281* gene amplified with P12 and P13 primer pair). Competitor DNA was added in 10-fold excess (molar ratio) over labeled fragment, unless indicated differently.

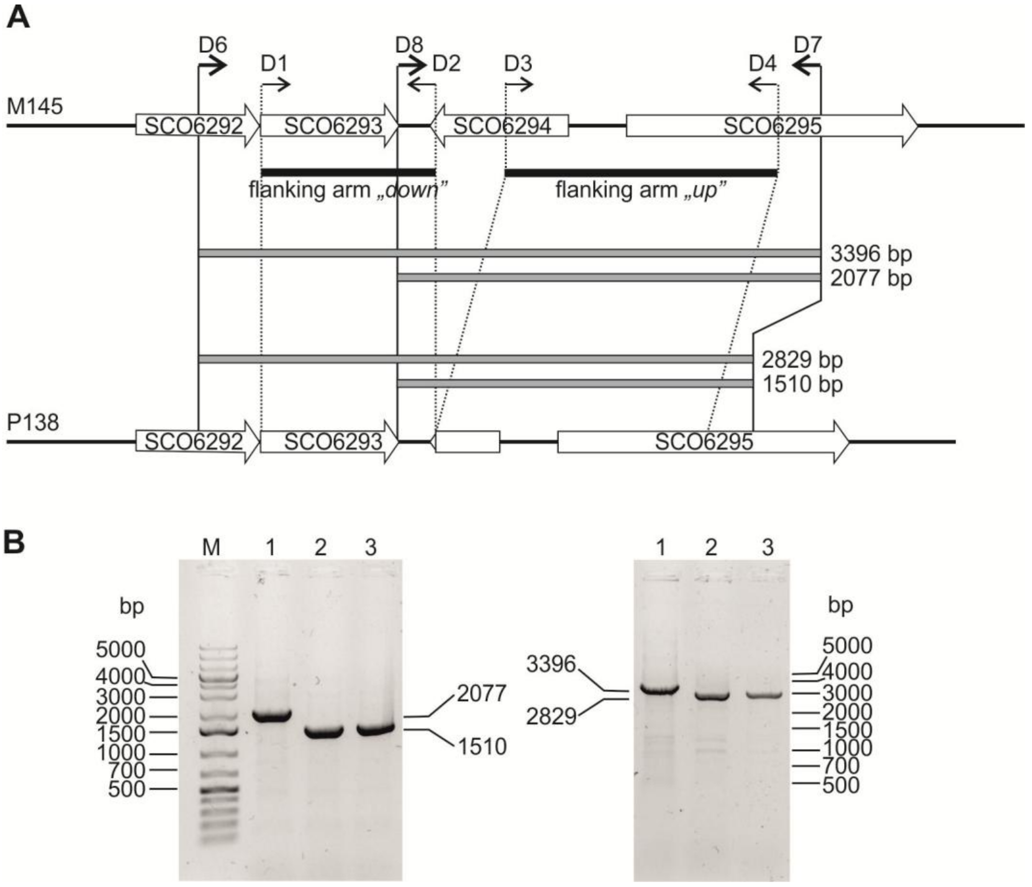

FIGURE S4 Deletion of *hypR (SCO6294)* gene and verification by PCR. **(A)** Location of primers and PCR products used as flanking arms for homologous recombination (black bars) and to verify deletion (grey bars), M145 – wild type, P138 – deletion mutant. **(B)** PCR products with primer pairs D8 and D7 (left panel) or D6 and D7 (right panel); 1 - M145, 2, 3 - P138 (two exconjugants), M - 1kb Plus DNA Ladder (Thermo Scientific)

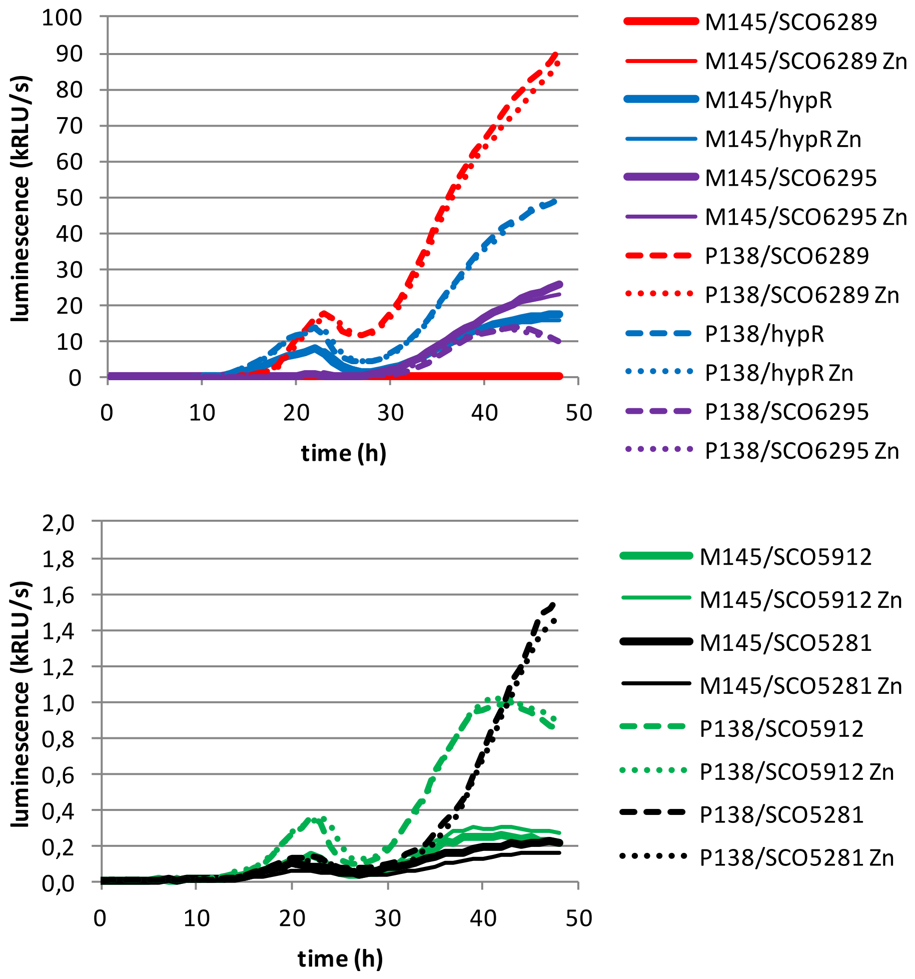

FIGURE S5 Activity of promoters measured as luminescence intensity in wild type (M145) and *hypR* deletion mutant (P138) strains carrying integrated promoter probe plasmids (pFLUXH derivatives) grown on solid modified 79 medium in the absence and presence of zinc ions (10 µM $ZnCl_2$). Measurements were taken in triplicate, graphs represent average values.

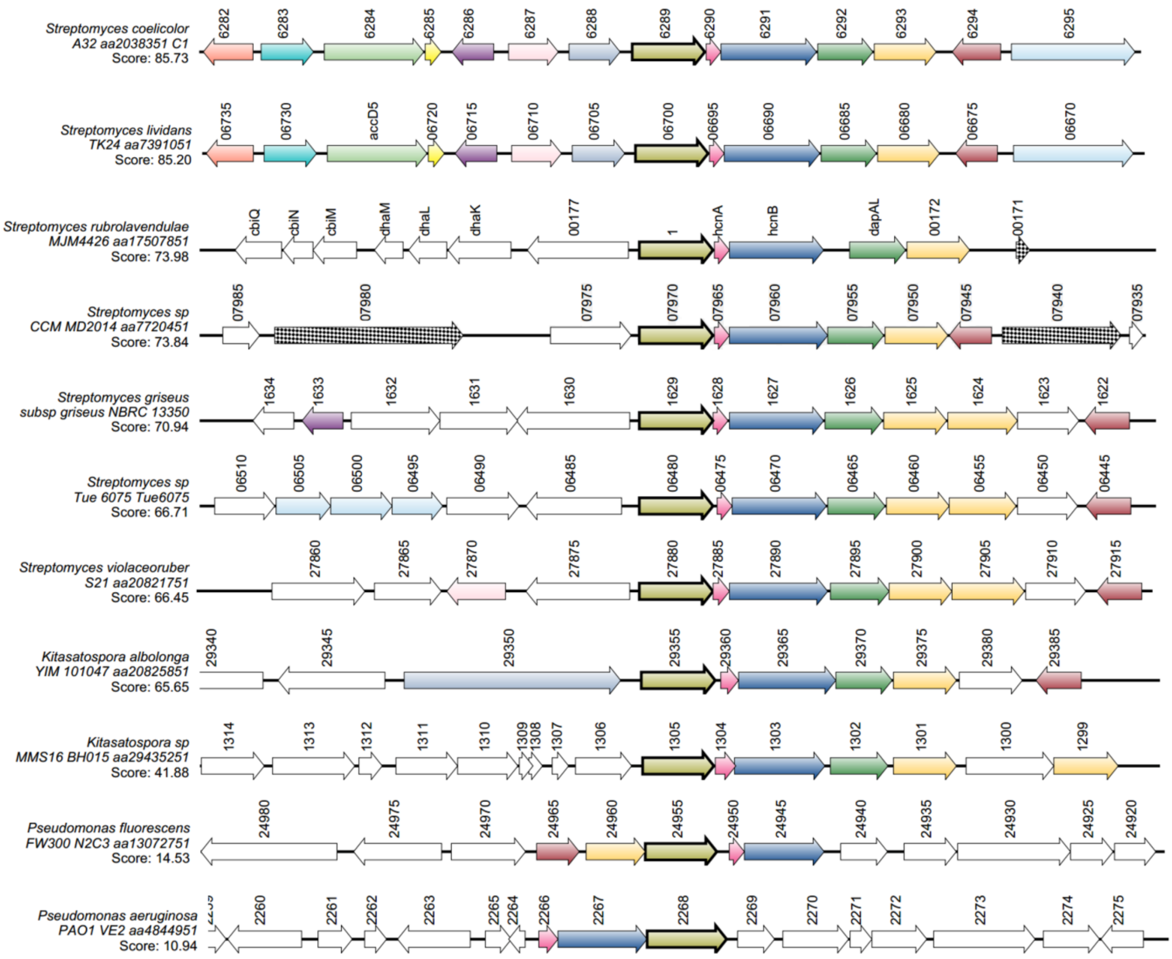

FIGURE S6 Synteny of *SCO6289-SCO6293* genes (predicted L-hydroxyproline utilization pathway) shared with *Streptomyces, Kitasatospora* and *Pseudomonas* genomes. SCO6289 amino acid sequence was used as a query for SyntTax (Oberto, BMC Bioinformatics, 14:4, 2013, doi: 10.1186/1471-2105-14-4).

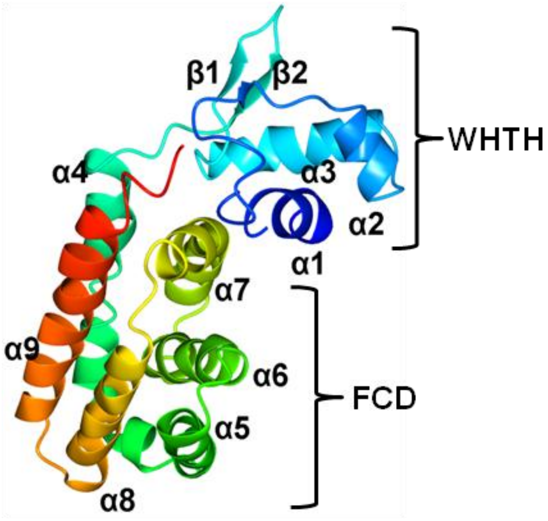

FIGURE S7 The overall architecture of HypR protein with DNA and ligand binding domains marked as WHTH and FCD, respectively. Model based on the crystal structure of TM0439 from *Thermotoga maritima* (PDB 3FMS) as a template was obtained with Phyre2 program (Kelley and Sternberg, Nat Protoc 4:363–373, 2009, doi: 10.1038/nprot.2009.2). TM0439 is a dimer, the model represents one subunit. Visualisation was made using CC4MP program version 2.10.6. The model is colored in blend through mode from blue to red, from N- to C-terminus, respectively.

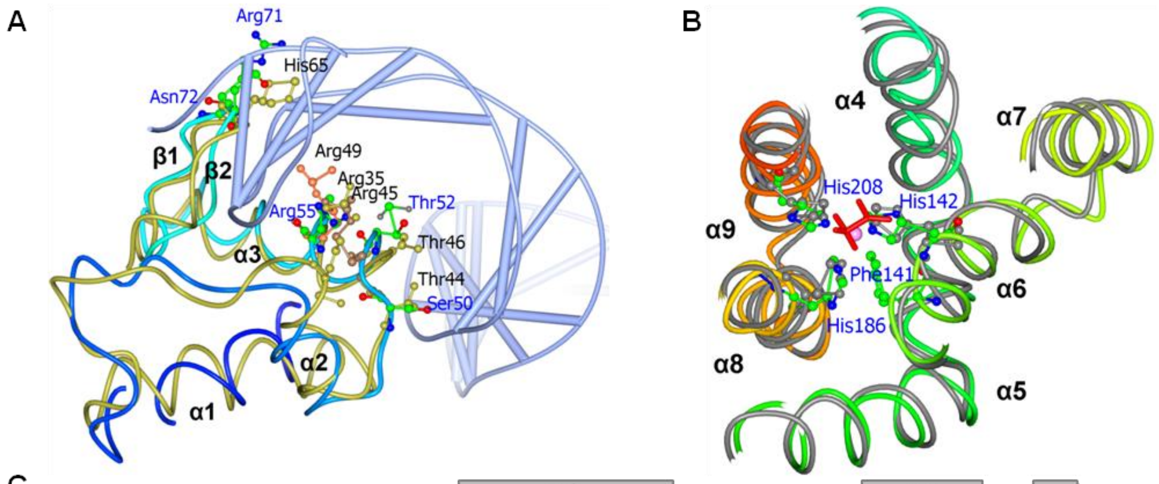

C

```
                α1  α2  α3  β1  β2  α4  α5  α6  α7  α8  α9
HypR   : ~MPHL----KSQAISVQTHLRDQVGDALRAALIAGDLRPGVVY-SAPTLAAELGVSATPV :  54
Tm0439 : ~MKKI---------E-VDLVRTKVYNLLKEMILNHELKLGEKL-NVRELSEKLGISFTPV :  48
CitO   : MNPTVEAVKRNLDLTSSKPLKICTYEAFKKTIILGDIPAGERI-NEKEFSEQLNISRTPI :  59
FadR   : ~~~~~~~~~~~~MVIKAQSPA-GFAEEYIIESIWNNRFPPGTILPAERELSELIGVTRTTL :  48

HypR   : REAMLDLAREGLVEAVRNKGFRVTEMTERDLDEFTEIRALIEVPTVGRVAAT-A------ : 107
Tm0439 : RDALLQLATEGLVKVVPRVGFFVTDVDEKFIRETIETRIMMEVFCLENYFDKIA------ : 102
CitO   : RFALQELVKEQLVEHIPMVGIVVKGISMKDAYEIYDIRKSLDTLATIKAMEL-M------ : 112
FadR   : REVLQRLARDGWLTIQHGKPTKVNNFWETSGLNILETLARLDHESVPQLIDNLLSVRTNI : 108

HypR   : ----TAEQLEALRPLAQQIVTAARKHDVLKYLEADHRFHLELL----ALAGNHHLVEVVA : 159
Tm0439 : ----GSEELLE---IKGEIDDVEKSAKREIFDDSDERLHKLFI----RASGNELIISLYE : 151
CitO   : ----TPEDFDELEALLLEGEQYNKNNQVDDLLQNFSDFNSFIY----TKSQMLRLKKIVT : 164
FadR   : STIFIRTAFRQHPDKAQ--EVLATANEVADHADAFAELDYNIFRGLAFASGNPIYGLILN : 166

HypR   : DLRKRSRLFGLGSLNETGRLVASAQEHVDLLDLMAAGRGGEAEECMRRHLAHVR-----T : 214
Tm0439 : KIWDRIDLVR--H--LNERYVVSNREHKELIERIISGDKEGAIEKLKEHLKNVEAETIKN : 207
CitO   : DLHAYLIYFRDIAIRASERRSIALEEHWLIFRGMKTKNIDQITLLTHEHLNRSLQF---I : 221
FadR   : GMKGLYTRIGRHYFANPEARSLALGFYHKLSALCSEGAHDQVYETVRRYGHES-----GE : 221

HypR   : LWADGRTNAED~~~~~~~ : 225
Tm0439 : LYTYERS~~~~~~~~~~~ : 214
CitO   : LKEMERRQIE~~~~~~~~ : 231
FadR   : IWHRMQKNLPGDLAIQGR : 239
```

FIGURE S8 Model of HypR based on the crystal structure of TM0439 from *Thermotoga maritima* (PDB 3FMS) as a template obtained with Phyre2 program (Kelley and Sternberg, Nat Protoc 4:363–373, 2009, doi: 10.1038/nprot.2009.2) **(A, B)** and alignment of HypR amino acid sequence with TM0439 and two other proteins from FadR subfamily, for which structural or functional data are available **(C)**. Visualisation was made using CC4MP program version 2.10.6. The model is colored in blend through mode from blue to red, from N- to C-terminus, respectively. Amino acids of HypR are numbered according to the corrected start codon. **(A)** DNA binding domain. The DNA was modeled by superposition of the FadR-DNA complex (PDB 1hw2) onto the WHTH domain of HypR. The structure of FadR is shown in gold and amino acids involved in DNA binding are shown in single colors and labeled in black. Putative DNA binding residues of HypR are colored according to atom type and labeled in blue. **(B)** Superposition of HypR and TM0439 FCD domains. TM0439 is shown in

grey together with His residues coordinating Ni^{2+} ion represented by a pink ball. Acetate molecule found in TM0439 structure is shown as red sticks. Histidines implicated in metal ion binding and Phe141 potentially involved in shaping of the ligand binding pocket in HypR are colored according to atom type and labeled in blue. **(C)** NCBI accession numbers: HypR – amino acids 39-263 of NP_630392, TM0439 from *Thermotoga maritima* – AGL49361, CitO from *Enterococcus faecalis* – ADX78673, FadR from *Escherichia coli* - WP_000234823. Predicted secondary structure elements of HypR protein are shown above the sequence. Black dots indicate amino acids of FadR interacting with DNA (Xu *et al.*, J Biol Chem 276:17373–17379, 2001). White circles and black arrowheads indicate amino acids of HypR predicted to bind DNA and metal ion, respectively. Asterisks show conserved amino acids which are important for ligand binding and correct function of FCD domain in CitO (Blancato *et al.*, Front Microbiol 7:2–5, 2016, doi: 10.3389/fmicb.2016.00101).
